# Supplementary figures and images for: Spontaneous peripheral oxygen desaturation and apnea events in mice vary by strain and inspired oxygen level
Source: bioRxiv. 2025 Sep 5:2025.09.01.673515. Preprint. [Version 1] doi: 10.1101/2025.09.01.673515 (PMC12424765; doi:10.1101/2025.09.01.673515)

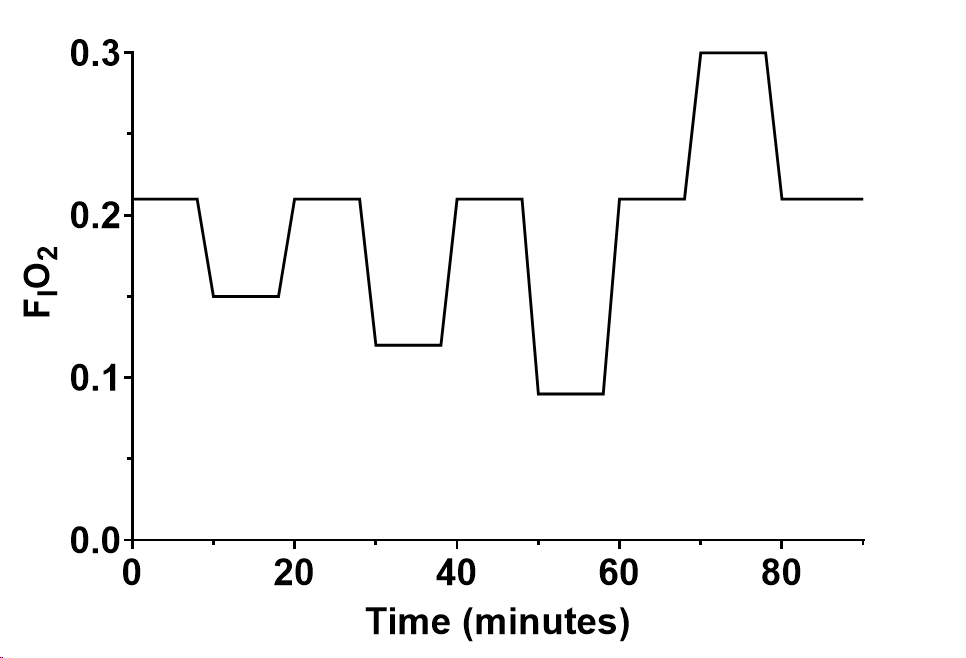

Supplement: Supplement 1 [file media-1.docx]
